# *catena*-Poly[cadmium(II)-(μ-3-ammonio-3-phenyl­propanoato-κ^2^ *O*:*O*′)-di-μ-chlorido]

**DOI:** 10.1107/S1600536808023179

**Published:** 2008-07-31

**Authors:** Zhi-Rong Qu, Xiu-Zhi Li

**Affiliations:** a Ordered Matter Science Research Center, College of Chemistry and Chemical Engineering, Southeast University, Nanjing 210096, People’s Republic of China

## Abstract

The title compound, [CdCl_2_(C_9_H_11_NO_2_)]_*n*_, is a coordination polymer prepared by the hydro­thermal reaction of cadmium(II) chloride and 3-amino-3-phenyl­propanoic acid. Geometric parameters are in the usual ranges. The cadmium cation is octa­hedrally coordinated by four Cl atoms at equatorial sites and two O atoms from two ligands at the axial sites. The material is composed of one-dimensional extended polymeric chains in which two Cl atoms bridge Cd atoms. The crystal structure is stabilized by an intra­molecular N—H⋯O hydrogen bond.

## Related literature

For related literature, see: Arki *et al.* (2004 ▶); Cohen *et al.* (2002 ▶); Zeller *et al.* (1965 ▶); Zhao (2007 ▶); Qu *et al.* (2004 ▶).
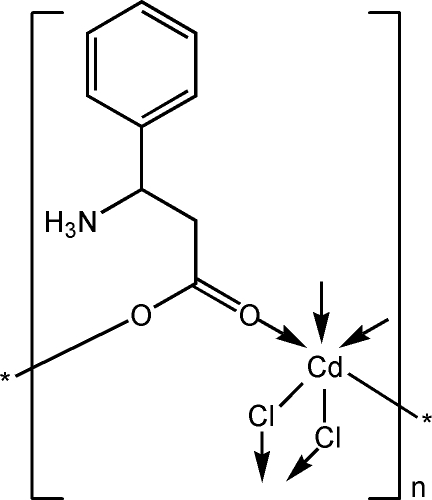


## Experimental

### 

#### Crystal data

[CdCl_2_(C_9_H_11_NO_2_)]
*M* *_r_* = 348.49
Monoclinic, 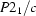

*a* = 11.879 (2) Å
*b* = 6.9364 (14) Å
*c* = 14.072 (3) Å
β = 110.26 (3)°
*V* = 1087.7 (4) Å^3^
*Z* = 4
Mo *K*α radiation
μ = 2.48 mm^−1^
*T* = 293 (2) K
0.25 × 0.18 × 0.15 mm

#### Data collection

Rigaku SCXmini diffractometer
Absorption correction: multi-scan (*CrystalClear*; Rigaku, 2005 ▶) *T*_min_ = 0.592, *T*_max_ = 0.690
9829 measured reflections
2148 independent reflections
1963 reflections with *I* > 2σ(*I*)
*R*_int_ = 0.029

#### Refinement

*R*[*F* ^2^ > 2σ(*F* ^2^)] = 0.018
*wR*(*F* ^2^) = 0.041
*S* = 0.92
2148 reflections
132 parameters
H-atom parameters constrained
Δρ_max_ = 0.29 e Å^−3^
Δρ_min_ = −0.31 e Å^−3^

### 

Data collection: *CrystalClear* (Rigaku, 2005 ▶); cell refinement: *CrystalClear*; data reduction: *CrystalClear*; program(s) used to solve structure: *SHELXS97* (Sheldrick, 2008 ▶); program(s) used to refine structure: *SHELXL97* (Sheldrick, 2008 ▶); molecular graphics: *SHELXTL* (Sheldrick, 2008 ▶); software used to prepare material for publication: *PRPKAPPA* (Ferguson, 1999 ▶).

## Supplementary Material

Crystal structure: contains datablocks I, global. DOI: 10.1107/S1600536808023179/bx2165sup1.cif

Structure factors: contains datablocks I. DOI: 10.1107/S1600536808023179/bx2165Isup2.hkl

Additional supplementary materials:  crystallographic information; 3D view; checkCIF report

## Figures and Tables

**Table 1:** Hydrogen-bond geometry (Å, °)

*D*—H⋯*A*	*D*—H	H⋯*A*	*D*⋯*A*	*D*—H⋯*A*
N1—H1*C*⋯O1	0.89	2.08	2.735 (3)	130

**Fig. 1.**
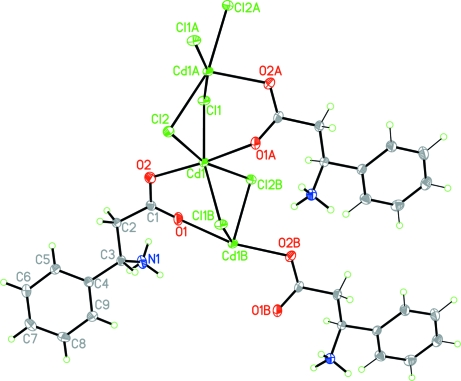
A partial packing diagram of the title compound, with the displacement ellipsoids were drawn at the 30% probability level. [Symmetry codes: (A) -x+1, y - 1/2, -z + 3/2; (B) -x + 1, y + 1/2, z + 3/2.]

**Fig. 2.**
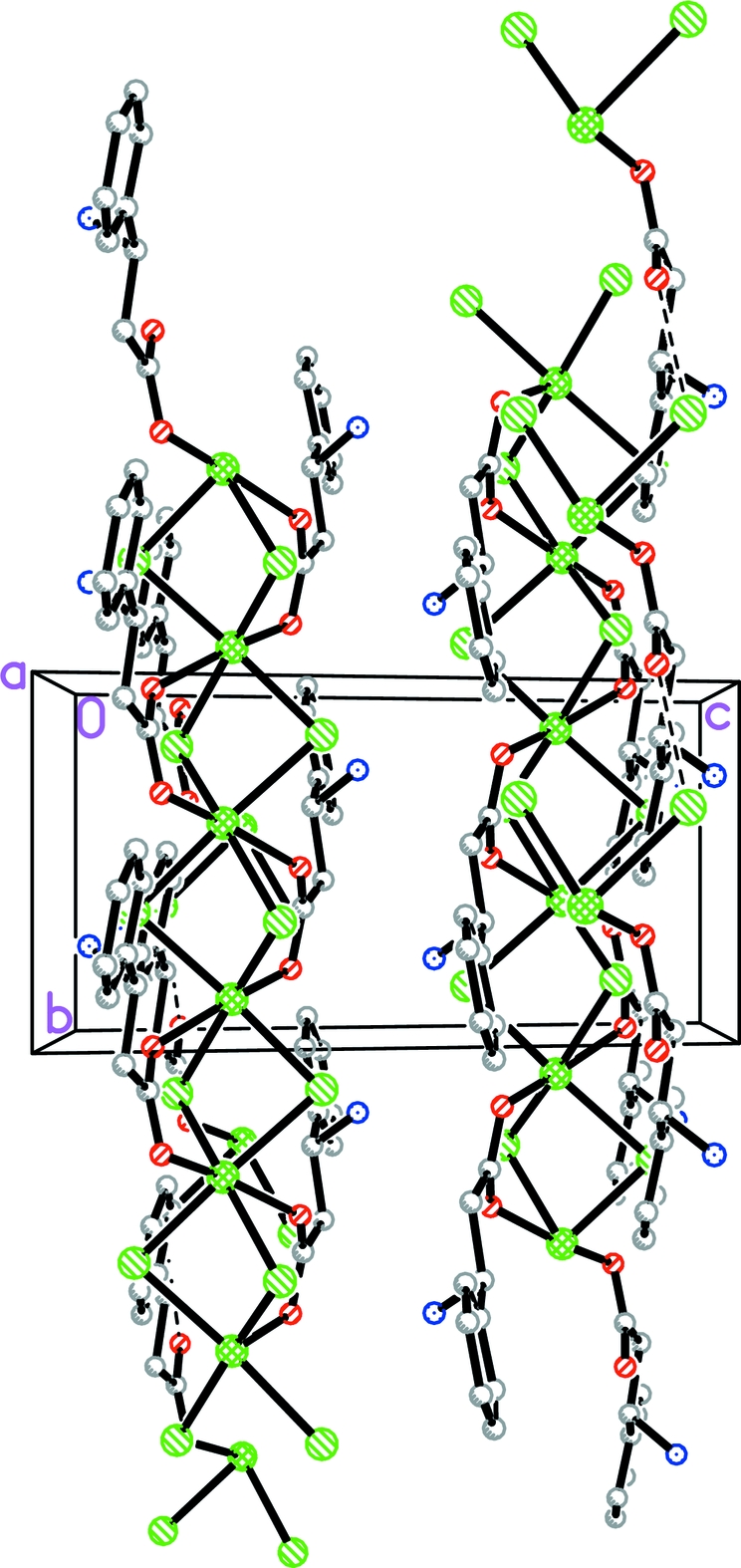
Packing diagram of the title compound, showing the structure along the b axis. H atoms have been omitted for clarity.

## supplementary crystallographic information

### Comment

Coordination frameworks have received much attention over the past decade because of their potential applications. β-amino acids are important molecules due to their pharmacological properties. Recently, there is an increased interest in the enantiomeric preparation of β-amino acids as precursors for the synthesis of novel biologically active compounds (Arki *et al.*, 2004; Cohen *et al.*, 2002; Zeller *et al.*, 1965). We report here the crystal structure of the title compound, which was obtained by the hydrothermal reaction of cadmium chloride and 3-amino-3-phenylpropanoic acid. In the structure of the title compoud, The geometric parameters are in the usual ranges (Zhao, 2007; Qu *et al.*, 2004). The cadmium cation is octahedrally coordinated by four Cl atoms at equatorial sites and two O atoms from two ligands at at axial sites (Fig. 1). The material is composed of one-dimensional extended polymeric chains in which two Cl atoms bridges Cd atoms (Fig. 2). The crystal structure is stabilized by an intramolecular hydrogen bond, (Table 1).

### Experimental

A mixture of CdCl_2_ (0.2 mmol, 0.037 g) and 3-amino-3-phenylpropanoic acid (0.2 mmol, 0.033 g) in H_2_O (4 ml) was heated in Pyrex tube at 100°C for two days. After slowly cooling down to room temperature over a period of 12 h, colorless crystals of the title compound suitable for diffraction were isolated.

### Refinement

Positional parameters of all the H atoms were calculated geometrically and were allowed to ride on the C, N atoms to which they are bonded. C—H = 0.97–0.98 Å, with 1.5*U*_eq_(methyl), C—H = 0.93 Å with *U*_iso_(H) = 1.2*U*_eq_(Caromatic) and N—H = 0.89 Å with *U*_iso_(H) = 1.5*U*_eq_(N).

### Crystal data

**Table:**

[CdCl_2_(C_9_H_11_NO_2_)]	*F*_000_ = 680
*M**_r_* = 348.49	*D*_x_ = 2.128 Mg m^−^^3^
Monoclinic, *P*2_1_/*c*	Mo *K*α radiation λ = 0.71073 Å
Hall symbol: -p 2ybc	Cell parameters from 1979 reflections
*a* = 11.879 (2) Å	θ = 3.1–27.5º
*b* = 6.9364 (14) Å	µ = 2.48 mm^−^^1^
*c* = 14.072 (3) Å	*T* = 293 (2) K
β = 110.26 (3)º	Prism, colourless
*V* = 1087.7 (4) Å^3^	0.25 × 0.18 × 0.15 mm
*Z* = 4	

### Data collection

**Table:**

Rigaku SCXmini diffractometer	2148 independent reflections
Radiation source: fine-focus sealed tube	1963 reflections with *I* > 2σ(*I*)
Monochromator: graphite	*R*_int_ = 0.029
Detector resolution: 13.6612 pixels mm^-1^	θ_max_ = 26.0º
*T* = 293(2) K	θ_min_ = 3.1º
CCD Profile fitting scans	*h* = −14→14
Absorption correction: multi-scan(CrystalClear; Rigaku, 2005)	*k* = −8→8
*T*_min_ = 0.592, *T*_max_ = 0.690	*l* = −17→17
9829 measured reflections	

### Refinement

**Table:**

Refinement on *F*^2^	Hydrogen site location: inferred from neighbouring sites
Least-squares matrix: full	H-atom parameters constrained
*R*[*F*^2^ > 2σ(*F*^2^)] = 0.018	*w* = 1/[σ^2^(*F*_o_^2^) + (0.0175*P*)^2^ + 1.2054*P*] where *P* = (*F*_o_^2^ + 2*F*_c_^2^)/3
*wR*(*F*^2^) = 0.041	(Δ/σ)_max_ = 0.001
*S* = 0.92	Δρ_max_ = 0.29 e Å^−^^3^
2148 reflections	Δρ_min_ = −0.31 e Å^−^^3^
132 parameters	Extinction correction: SHELXL97 (Sheldrick, 2008), Fc^*^=kFc[1+0.001xFc^2^λ^3^/sin(2θ)]^-1/4^
Primary atom site location: structure-invariant direct methods	Extinction coefficient: 0.0014 (2)
Secondary atom site location: difference Fourier map	

### Special details

**Table:**

Geometry. All e.s.d.'s (except the e.s.d. in the dihedral angle between two l.s. planes) are estimated using the full covariance matrix. The cell e.s.d.'s are taken into account individually in the estimation of e.s.d.'s in distances, angles and torsion angles; correlations between e.s.d.'s in cell parameters are only used when they are defined by crystal symmetry. An approximate (isotropic) treatment of cell e.s.d.'s is used for estimating e.s.d.'s involving l.s. planes.
Refinement. Refinement of *F*^2^ against ALL reflections. The weighted *R*-factor *wR* and goodness of fit *S* are based on *F*^2^, conventional *R*-factors *R* are based on *F*, with *F* set to zero for negative *F*^2^. The threshold expression of *F*^2^ > σ(*F*^2^) is used only for calculating *R*-factors(gt) *etc*. and is not relevant to the choice of reflections for refinement. *R*-factors based on *F*^2^ are statistically about twice as large as those based on *F*, and *R*- factors based on ALL data will be even larger.

### Fractional atomic coordinates and isotropic or equivalent isotropic displacement parameters (Å^2^)

**Table:**

	*x*	*y*	*z*	*U*_iso_*/*U*_eq_	
Cd1	0.523335 (14)	0.11166 (2)	0.744199 (12)	0.02328 (7)	
Cl1	0.40439 (5)	−0.13994 (8)	0.60907 (4)	0.02792 (13)	
Cl2	0.67267 (5)	−0.16544 (8)	0.83106 (4)	0.02717 (13)	
O1	0.57035 (16)	0.4852 (3)	0.64381 (14)	0.0390 (4)	
O2	0.64928 (15)	0.1924 (2)	0.65962 (13)	0.0336 (4)	
N1	0.66195 (17)	0.7705 (3)	0.55775 (16)	0.0339 (5)	
H1A	0.6566	0.7490	0.4940	0.051*	
H1B	0.6696	0.8964	0.5705	0.051*	
H1C	0.5959	0.7275	0.5670	0.051*	
C1	0.6496 (2)	0.3701 (3)	0.64067 (17)	0.0248 (5)	
C2	0.7528 (2)	0.4489 (3)	0.61134 (18)	0.0261 (5)	
H2A	0.7376	0.4211	0.5404	0.031*	
H2B	0.8263	0.3834	0.6508	0.031*	
C3	0.76996 (19)	0.6662 (3)	0.62857 (17)	0.0238 (5)	
H3	0.7715	0.6913	0.6975	0.029*	
C4	0.8836 (2)	0.7527 (3)	0.62093 (17)	0.0243 (5)	
C5	0.9706 (2)	0.6450 (3)	0.6001 (2)	0.0315 (5)	
H5	0.9576	0.5147	0.5847	0.038*	
C6	1.0771 (2)	0.7308 (4)	0.6019 (2)	0.0373 (6)	
H6	1.1362	0.6563	0.5903	0.045*	
C7	1.0959 (2)	0.9252 (4)	0.62066 (19)	0.0359 (6)	
H7	1.1661	0.9831	0.6198	0.043*	
C8	1.0096 (2)	1.0326 (4)	0.6407 (2)	0.0355 (6)	
H8	1.0217	1.1640	0.6533	0.043*	
C9	0.9049 (2)	0.9479 (4)	0.64233 (19)	0.0318 (5)	
H9	0.8483	1.0219	0.6578	0.038*	

### Atomic displacement parameters (Å^2^)

**Table:**

	*U*^11^	*U*^22^	*U*^33^	*U*^12^	*U*^13^	*U*^23^
Cd1	0.02816 (10)	0.01635 (9)	0.02832 (10)	−0.00091 (7)	0.01356 (7)	−0.00123 (6)
Cl1	0.0328 (3)	0.0208 (3)	0.0266 (3)	0.0000 (2)	0.0057 (2)	0.0004 (2)
Cl2	0.0264 (3)	0.0230 (3)	0.0326 (3)	0.0018 (2)	0.0108 (2)	0.0014 (2)
O1	0.0391 (10)	0.0336 (10)	0.0582 (12)	0.0027 (8)	0.0345 (9)	0.0042 (9)
O2	0.0390 (10)	0.0263 (9)	0.0436 (10)	−0.0021 (8)	0.0244 (8)	0.0053 (8)
N1	0.0275 (10)	0.0356 (12)	0.0424 (12)	0.0052 (9)	0.0169 (9)	0.0133 (10)
C1	0.0291 (12)	0.0263 (12)	0.0220 (11)	−0.0050 (10)	0.0127 (9)	−0.0003 (9)
C2	0.0280 (12)	0.0224 (11)	0.0337 (13)	−0.0031 (9)	0.0182 (10)	−0.0014 (10)
C3	0.0250 (11)	0.0238 (11)	0.0255 (11)	0.0017 (9)	0.0127 (9)	0.0026 (9)
C4	0.0251 (11)	0.0251 (12)	0.0246 (11)	−0.0017 (9)	0.0110 (9)	0.0022 (9)
C5	0.0333 (13)	0.0250 (12)	0.0421 (14)	−0.0014 (10)	0.0205 (11)	−0.0035 (11)
C6	0.0284 (13)	0.0446 (16)	0.0444 (15)	0.0016 (12)	0.0195 (11)	−0.0013 (13)
C7	0.0278 (12)	0.0443 (16)	0.0344 (14)	−0.0108 (11)	0.0095 (11)	0.0023 (12)
C8	0.0366 (14)	0.0259 (12)	0.0401 (15)	−0.0078 (11)	0.0081 (11)	−0.0009 (11)
C9	0.0311 (13)	0.0267 (12)	0.0378 (14)	0.0016 (10)	0.0123 (11)	−0.0028 (11)

### Geometric parameters (Å, °)

**Table:**

Cd1—O2	2.2807 (17)	C2—C3	1.529 (3)
Cd1—O1^i^	2.3888 (18)	C2—H2A	0.9700
Cd1—Cl1^ii^	2.5965 (8)	C2—H2B	0.9700
Cd1—Cl1	2.6072 (8)	C3—C4	1.515 (3)
Cd1—Cl2	2.6141 (8)	C3—H3	0.9800
Cd1—Cl2^ii^	2.6880 (8)	C4—C5	1.386 (3)
Cl1—Cd1^i^	2.5965 (8)	C4—C9	1.391 (3)
Cl2—Cd1^i^	2.6880 (8)	C5—C6	1.391 (3)
O1—C1	1.246 (3)	C5—H5	0.9300
O1—Cd1^ii^	2.3888 (18)	C6—C7	1.377 (4)
O2—C1	1.261 (3)	C6—H6	0.9300
N1—C3	1.509 (3)	C7—C8	1.373 (4)
N1—H1A	0.8900	C7—H7	0.9300
N1—H1B	0.8900	C8—C9	1.383 (3)
N1—H1C	0.8900	C8—H8	0.9300
C1—C2	1.524 (3)	C9—H9	0.9300
			
O2—Cd1—O1^i^	166.93 (6)	C1—C2—H2A	109.0
O2—Cd1—Cl1^ii^	98.98 (5)	C3—C2—H2A	109.0
O1^i^—Cd1—Cl1^ii^	79.65 (5)	C1—C2—H2B	109.0
O2—Cd1—Cl1	94.10 (5)	C3—C2—H2B	109.0
O1^i^—Cd1—Cl1	88.67 (5)	H2A—C2—H2B	107.8
Cl1^ii^—Cd1—Cl1	166.089 (9)	N1—C3—C4	109.76 (18)
O2—Cd1—Cl2	87.88 (5)	N1—C3—C2	109.37 (19)
O1^i^—Cd1—Cl2	79.47 (5)	C4—C3—C2	116.91 (19)
Cl1^ii^—Cd1—Cl2	97.59 (3)	N1—C3—H3	106.8
Cl1—Cd1—Cl2	87.56 (3)	C4—C3—H3	106.8
O2—Cd1—Cl2^ii^	106.76 (5)	C2—C3—H3	106.8
O1^i^—Cd1—Cl2^ii^	86.18 (5)	C5—C4—C9	118.5 (2)
Cl1^ii^—Cd1—Cl2^ii^	86.24 (3)	C5—C4—C3	123.3 (2)
Cl1—Cd1—Cl2^ii^	85.47 (3)	C9—C4—C3	118.0 (2)
Cl2—Cd1—Cl2^ii^	164.176 (16)	C4—C5—C6	120.4 (2)
Cd1^i^—Cl1—Cd1	85.28 (3)	C4—C5—H5	119.8
Cd1—Cl2—Cd1^i^	83.32 (3)	C6—C5—H5	119.8
C1—O1—Cd1^ii^	142.04 (16)	C7—C6—C5	120.5 (2)
C1—O2—Cd1	113.60 (14)	C7—C6—H6	119.7
C3—N1—H1A	109.5	C5—C6—H6	119.7
C3—N1—H1B	109.5	C8—C7—C6	119.2 (2)
H1A—N1—H1B	109.5	C8—C7—H7	120.4
C3—N1—H1C	109.5	C6—C7—H7	120.4
H1A—N1—H1C	109.5	C7—C8—C9	120.8 (2)
H1B—N1—H1C	109.5	C7—C8—H8	119.6
O1—C1—O2	124.1 (2)	C9—C8—H8	119.6
O1—C1—C2	117.9 (2)	C8—C9—C4	120.5 (2)
O2—C1—C2	118.0 (2)	C8—C9—H9	119.8
C1—C2—C3	112.72 (19)	C4—C9—H9	119.8

Symmetry codes: (i) −*x*+1, *y*−1/2, −*z*+3/2; (ii) −*x*+1, *y*+1/2, −*z*+3/2.

### Hydrogen-bond geometry (Å, °)

**Table:**

*D*—H···*A*	*D*—H	H···*A*	*D*···*A*	*D*—H···*A*
N1—H1C···O1	0.89	2.08	2.735 (3)	130
